# Association between Malnutrition and Dental Caries in Iraqi Kurdish Children

**DOI:** 10.3390/dj11060141

**Published:** 2023-05-26

**Authors:** Mohammed Khalid Mahmood, Romain Lan, Herve Tassery, Delphine Tardivo

**Affiliations:** 1 Faculty of Dentistry, Aix-Marseille University, CNRS, EFS, ADES, 13284 Marseille, France; 2 Odontology Department, Timone Hospital, 13284 Marseille, France; 3 Ecole de Médecine Dentaire de Marseille, Université d’Aix-Marseille, 13385 Marseille, France

**Keywords:** children, dental caries, malnutrition, vitamin D

## Abstract

Background: This paper aimed to study the association of serum Vitamin D level and Body Mass Index (BMI) as representatives of malnutrition at micro and macro levels, respectively, on dental caries. Method and Participants: A total of 333 randomly selected children aged 6–12 years in Sulaimani, Kurdistan, Iraq were examined for three variables of the Decayed Missed Filled Tooth (DMFT) index, BMI, and Vitamin D serum levels in a single-time cross-sectional snapshot. Results: The majority of the population studied (70%) were Vitamin D deficient. In the linear regression analysis, both Vitamin D and BMI had insignificant effects on DMFT, with *p*-values of 0.22 and 0.55, respectively. After the categorization of the data, the risk estimate between normal (≥20 ng/mL) and deficient (<20 ng/mL) Vitamin D groups with regards to the caries and caries-free categories was 1.97 (95% CI: 0.91–4.24). According to the mean and median of the DMFT, which were both 4, the sample is categorized into the low-caries group (DMFT < 4) and high-caries group (DMFT > 4). When these groups are compared to Vitamin D levels and taking 20 and 15 as thresholds, the odds ratios were 1.19 (CI: 0.74–1.92) and 1.88 (CI: 1.20–2.94), respectively. Regarding the BMI, a normal BMI increases the chances of having a lower caries index (OR = 1.83, CI: 1.10–3.03). Conclusions: Our results show that having a serum Vitamin D level ≥15 ng/mL and having a normal BMI are associated with a lower caries index in children.

## 1. Introduction

With billions of people affected and a major impact on quality of life, dental caries is one of the most widespread diseases in the world. Tooth decay is a complex condition with various direct and indirect risk factors, primarily genetic and environmental. Identifying modifiable risk variables that could be the focus of efficient intervention is necessary to understand how hereditary and environmental risk factors affect dental caries [1].

Furthermore, the WHO reports that caries is the fourth most costly chronic condition to treat [2]. The pathogenesis of this infectious disease is diverse and multifactorial. The most extensively researched risk factors were environmental ones, including cariogenic diets high in carbohydrates, cariogenic microorganisms, and poor dental hygiene [3]. However, because some patients are more susceptible to or resistant to caries than others when exposed to the same environmental risk factors, environmental factors alone are insufficient to explain the prevalence and incidence of caries [4].

Malnutrition, on the other hand, is classified as an abnormal physiological condition and is defined as the inadequate, unbalanced, or excessive ingestion of macronutrients, micronutrients, or both. One of the most important health issues facing the globe today is malnutrition, which affects children’s growth, development, health, and overall well-being [5]. Malnutrition is primarily caused by inadequate food intake, severe and recurrent illnesses, or a combination of the two. Socioeconomic and demographic factors, such as parental occupation and education, marital status, family income, mothers’ nutritional knowledge, housing location (urban or rural), gender, and water supply, might have an impact on children’s nutritional status [6].

Malnutrition has an effect on dental health and poor oral health can lead to malnutrition. Good dental health is either promoted or prevented by this interdependent relationship and vice versa. Malnutrition may affect the body’s homeostasis, lowering resistance to microbial biofilm and reducing the ability for tissue healing, all of which can lead to dental caries. For instance, a reduction in resistance to microbial biofilm will make dental hard tissue more susceptible to decay [7], and a decrease in the ability of tissue healing is best reflected in the re-mineralization process of early enamel defects [8]. According to the suggestions of some studies, malnutrition may cause caries through processes such as enamel hypoplasia, decreased salivary gland activity, and altered saliva composition [9,10]. On the other hand, many studies have shown the impact of dental caries on malnutrition [11,12] and the effect of treating carious teeth on the improvement of nutrition status and quality of life in children has been proposed [13].

Malnutrition is broadly classified into two types:

1. Micro-nutrient-related malnutrition includes the deficiency or excess of important vitamins and minerals.
2. Age-specific weight- and height-related malnutrition [14].

Vitamins have an important impact on dental and overall health and their imbalance can cause malnutrition. Vitamin D is a known steroid hormone that can be obtained via diet and dietary supplements in addition to sunshine exposure [15]. Oily fish and oils from fish are the only rare foods that naturally contain vitamin D [16]. The terms vitamin D2 and vitamin D3 are referred to collectively as simply vitamin D. Vitamin D2 is produced by exposing yeast to ultraviolet light, which results in the production of ergosterol. Vitamin D3 is produced by exposing lanolin to ultraviolet light, which results in the production of 7-dehydrocholesterol. 7-dehydrocholesterol has the same biological action as cholecalciferol (vitamin D3) and is produced in human skin. The measurement of serum 25-hydroxyvitamin D (25[OH]D) is a biomarker that is commonly utilized for the purpose of evaluating vitamin D status [15].

As a significant micronutrient, vitamin D controls calcium levels and is crucial for craniofacial development and maintaining good oral health. Endogenous synthesis and exogenous acquisition via diet and supplements are the two main ways to get vitamin D. Sunlight, poor absorption, body mass index, age, place of residence, skin color, the season of the year, and vitamin D supplementation are known to have influences on vitamin D levels [16].

Epidemiological research suggests that vitamin D deficiency is an epidemic that is widespread, with more than half of the world’s population being at risk for the condition. [17].

Teeth are mineralized organs located at the alveolar bones of the jaws. The mineralization of teeth happens simultaneously with that of the skeleton, but if mineral metabolism is disrupted, tooth failures will resemble those that affect bone tissue. When vitamin D levels are not in equilibrium, it can cause faulty hypomineralization which makes teeth extremely prone to fracture and decay [18].

There is considerable discussion and debate about the methods by which Vitamin D deficiency (VDD) affects tooth mineralization in the existing literature [19]. The primary biological justification is based on the fact that severe VDD (10 ng/mL) results in secondary hyperparathyroidism, hypophosphatemia, and hypocalcemia. This hyperparathyroidism encourages intestinal absorption of calcium (Ca^2+^) and renal synthesis of 1,25[OH]2D, promoting bone turnover and resulting in elevated serum Ca^2+^ levels and decreased serum inorganic phosphate (Pi) levels [20,21]. The original hypophosphatemia then becomes significantly worse. As a consequence of this, correct mineralization of teeth is impeded, which results in mineralization anomalies. This is because the breakdown of vitamin D signaling pathways in tooth cells with insufficient quantities of Ca^2+^ and phosphate ions causes the pathways to break down [19].

Interestingly, maternal 25(OH)D levels can affect deciduous dentition despite the role of inherited fetal abnormalities [22,23]. Vitamin D levels in fetal serum reflect maternal levels and can be utilized as a common potential marker for the fetus [24]. Hence, if maternal 25(OH)D levels become out of equilibrium, this could directly affect the health of the baby [25] and, in particular, the development of their teeth [26,27,28]. The specific week of gestation in which the mother experienced VDD is what determines the pattern of mineralization deficiency that is present. For example, the human primary maxillary central incisor begins to calcify during the 13th week of pregnancy. However, if VDD is present, there may be a hypoplasia and/or mineralization deficit on the incisal third of the crown. This is because the human primary maxillary central incisor begins to calcify during the 13th week of pregnancy. It is now believed that maternal VDD manifests itself between the ages of 12 and 16 weeks, 20 and 32 weeks, and 36 and 40 weeks, respectively, resulting in anomalies in the incisal third, middle third, and cervical third, respectively. [28]. Vitamin D supplementation during gestation was studied in a randomized clinical trial (RCT), which found that pregnant women with vitamin D levels below 15 ng/mL had a 14% increased likelihood of dental caries development in the primary teeth of their children [28]. On the other hand, taking a vitamin D supplement in high doses during pregnancy was associated with a lower risk of enamel defects that was fifty percent lower [26,29]. An association between high-dose vitamin D supplementation throughout pregnancy and a 50% decreased incidence of enamel defects in the newborn is postulated in a controlled clinical experiment. This finding lends evidence to the potential significance of vitamin D as a prophylactic therapy for enamel defects [26]. Recently, a meta-analysis that included data from 2827 controlled clinical trials on the effect of vitamin D supplementation on preventing dental caries was examined. According to this study’s findings, supplementing with vitamin D decreased dental caries risk by roughly 47%, although this finding has a low certainty level [30].

Vitamin D levels have been investigated in relation to various aspects of oral health. A lack of Vitamin D causes osteoporosis and decreased bone density, leading to the spread of periodontal disease. On the other hand, adequate amounts of this vitamin, which functions as an immune-modulating, anti-inflammatory, and anti-proliferative agent, may lower the incidence of gingivitis and periodontitis [31]. In the early stages of development, vitamin insufficiency and nutritional status significantly impact the hard dental tissues [9]. In children, a link between malnutrition, enamel hypoplasia, and caries in primary dentition was proposed [32].

Moreover, teeth loss results in poorer dietary choices and weaker nutritional status [33]. Vitamin D levels significantly increased in partly dentate patients under the age of 65 who had their missing teeth replaced with prosthetics [34]. In general, higher serum levels of vitamin D (above 30 ng/mL) have been demonstrated to be connected with improved oral health outcomes [35]. To sum up, Vitamin D is associated with the two main oral diseases, dental caries and periodontitis [31,32,35].

It is vital to note that the primary structure of teeth is not radically altered by serum vitamin D since this structure is constant until an exogenous influence wears it down. However, vitamin D apparently prevents caries formation by regulating the immune system and encouraging bacteria eradication [36].

For the measurement of malnutrition at a macro level, besides the WHO’s international z-scores of height-for-age, weight-for-age, and weight-for-height, age-specific BMI is a practical and useful tool for providing insight into child growth, assessing malnutrition, and classifying children into underweight, normal, and overweight groups. BMI is an index of weight for height and is measured by dividing a person’s weight by the square of his/her height (kg/m^2^).

Regarding the association between BMI and dental caries, some papers suggest a positive correlation, while other results are negative and/or inconclusive [37,38,39]. Most of the published papers on this association focused on overweight and obese children compared to the underweight group, which is studied only in a few articles. This could be because of the relative clarity of the association between excess weight and dental caries. Obesity, through higher carbohydrate consumption and snack habits between meals, promotes demineralization and the caries production process [40]. In contrast, for the underweight group and malnutrition represented by a lower age-specific BMI, it may be postulated that lack of intake of necessary elements and minerals may result in a tooth content that is more prone and less resistant to dental caries [41].

There are several types of research of different study designs and of various significance levels on the association between malnutrition and dental caries worldwide. However, there is a knowledge gap regarding the direction and extent of this association among Iraqi children specifically. Hence, this paper aimed to study this association by taking Vitamin D serum level and BMI as representatives of malnutrition at micro and macro levels.

## 2. Subjects and Methods

### 2.1. Study Design and Ethical Considerations

Children aged 6–12 years were examined for the studied variables in a single-time cross-sectional snapshot. Ethical approval and the Institutional Review Board’s consent were taken from Sulaimani University (no: 213/2022).

### 2.2. Participants

Participants were recruited from the social media accounts of Harem Hospital in Sulaimani, Kurdistan, Iraq, announcing the availability of free dental check-ups, serum Vitamin D assessment, and BMI screening for children. Only apparently healthy children were included in the survey. Children suffering from endocrine diseases like diabetes mellitus, malabsorption problems like celiac disease and lactose intolerance, and children with special needs were excluded. Parents were informed about the nature of the research’s content and their consent was taken. 

### 2.3. Sample Size

Calculation of the minimum and effective sample size is found by using Kish’s [42] formula as follows: N = (Zα/2)^2^ (P) (1 − P)/D^2^, where N is the sample size, Zα/2 is the standard normal value at 95% which is CI = 1.96, D (delta) is the precision of 5% or the marginal error (in this study, we took it as 0.05). P stands for the prevalence of the studied variable in previous articles at the same location. Previous research performed in Iraq estimated the expected prevalence of malnutrition to be 15% [43]. To be on the safe side, we set P at 25%:

N = (1.96)^2^ (0.25) (1 − 0.25)/(0.05)^2^ = 288

Thus, the minimum sample size is 288. Nonetheless, the sample size of this paper included 333 participants.

### 2.4. Study Measures and Data Collection

Children were examined for three variables: DMFT index, BMI, and Vitamin D serum levels.

The DMFT index is a measurement for dental caries ranging from 0–28 for permanent dentition and 0–20 for primary dentition, in which 0 means no caries, and the higher the number, the higher the number of dental caries. DMFT and dmft are used to assess the permanent and primary dentitions respectively and their average is calculated for mixed dentitions. However, for easier presentation and understanding, only the notation of DMFT is used in the tables and in the text. Each tooth is given a single score. A healthy, non-carious, not filled, and not missing due to caries tooth is given a score of 0. In contrast, a carious tooth, filled with restoration or missing due to caries, is given a score of 1. Next, the DMFT Index is calculated by adding the scores of all teeth. The examination is done by two calibrated examiners on dental chairs using a mirror and probe under sufficient artificial light.

For the purpose of further comparison and according to the mean and median of the population studied which were both 4, the sample is categorized into the low caries group (DMFT = 0–4) and the high caries group (DMFT = 5–14). This type of categorization was frequently done in previous papers studying the association [44,45].

Blood samples were collected from the participants to assess serum Vitamin D by one of the most widely used tests which is the measurement of 25-hydroxyvitamin D (25[OH]D) in the serum. A level ≥20 ng/mL, which is proposed by the American Endocrine Society, is taken as a normal value. In addition, according to the mean and median of the studied population, which were 16 and 15, respectively, ≥15 ng/mL was set as a sufficient threshold. This value almost corresponds to the Institute of Medicine’s perspective on Vitamin D sufficiency for an overall healthy individual [46]. Out of the participants, 206 (61.7%) of the cases were collected in summer and the rest of the sample, comprising 128 cases (38.3%), was gathered in winter.

The body weight and height of children were measured using standardized methods and equipment, as recommended in the literature [47]. Next, the age-specific online BMI calculator from the Centers for Disease Control and Prevention (CDC) is used to calculate the BMI. Despite the fact that z-scores of weight-for-age and height-for-age are golden standards to diagnose child malnutrition, we used the more practical age-specific BMI, since it gives a single numerical value and contains all the components of weight, height, age, and gender. Furthermore, it is one of the measures recommended by the WHO in malnutrition assessment [48]. In addition, the underweight and overweight categories were treated as abnormal and compared to their normal BMI counterpart.

### 2.5. Data Analysis

Multivariate regression analysis is performed to investigate the effects of Vitamin D level, BMI, and other possible confounders on DMFT. Furthermore, DMFT, Vitamin D, and BMI variables were categorized to assess odds ratios between the categories. Categorical analysis is common in the literature to study these associations [49,50,51]. This is probably due to the high number of known and unknown covariates affecting the correlation.

Statistical analysis was performed via the Statistical Package for the Social Sciences (SPSS) (SPSS for Windows, Version 25.0, Chicago, IL, USA) program. Numerical variables were presented as the median and interquartile range (IQR), while categorical variables were presented as the frequency and percentage. Normal distribution was evaluated by the Kolmogorov–Smirnov test. Numerical data were compared between groups using the Mann–Whitney U test. The crude odds ratio was used to estimate the risk estimate of different categories. A *p*-value of <0.05 was considered significant.

## 3. Results

A total of 333 children were included in the research (188 females and 146 males). Participants’ median age was eight, ranging from 6–12. Table 1 shows the descriptive statistics of the participants.

Most children (70%) were found to be Vitamin D deficient (mean = 16.6, median = 15). Even when ≥15 ng/mL is considered a sufficient borderline, half of the population was still deficient (49.5%).

Regarding the BMI, ~30% of the cases had an abnormal BMI, of which 18.3% were overweight and 11.4% were underweight.

Most children had at least one carious tooth (85.9%), while 47 cases were caries-free (14.1%). The maximum number for DMFT was 14. Both the median and mean of the DMFT score were 4. Furthermore, the score of 4 is taken as a borderline to categorize DMFT into two subcategories: Low caries, which contained DMFT scores 0–4 (n = 203, 61%), and High caries which included DMFT scores 5–14 (n = 130, 39%).

The comparison between the sexes revealed higher levels of Vitamin D concentration and dental caries in boys (Table 2). In contrast, age and BMI were not significantly different among the two groups.

A comparison of the three main variables according to the season in which the data were collected revealed that Vitamin D levels were significantly higher in summer in contrast to winter. On the other hand, the BMI was higher in winter than in summer (Table 3).

Taking DMFT as the dependent variable, multivariate regression analysis was done to check the effects of age, sex, seasons, BMI, and vitamin D on DMFT. Age was the only significant variable after adjustment (Table 4). Age and BMI negatively affected DMFT, while vitamin D levels were positively associated with the DMFT index.

Next, the data are categorized to examine the crude ORs. When Vitamin D is categorized into normal concentration (≥20 ng/mL) versus deficient (<20 ng/mL) and DMFT is classified into caries and caries-free categories, the odds ratio was 1.97 (95% CI: 0.91–4.24). Although the exact OR suggests that a child with a normal Vitamin D serum level has a 97% greater chance to be caries-free compared to a Vitamin D-deficient child, the wide CI ranges, which include 0, reduce the effect (Table 5).

Similarly, when a concentration ≥15 ng/mL is regarded as a sufficient threshold for Vitamin D and DMFT is categorized into caries and caries-free categories, the OR was 1.60 with a wide 95% CI range (0.85–3) (Table 5).

When comparing the low- and high-caries groups, in the first scenario, when the standard (≥20 ng/mL) was tested, the difference between the two groups was not significant: OR = 1.19 (CI: 0.74–1.92). However, when ≥15 ng/mL is taken as a threshold, there is a 88% higher chance on average for children with Vitamin D concentration ≥15 ng/mL to have a lower DMFT score as compared to the <15 ng/mL group (OR = 1.88, CI: 1.20–2.94) (Table 6).

Regarding the relationship between BMI and dental caries, having a normal BMI is not significantly associated with an increase in the chances of having no dental caries (OR = 1.12, CI: 0.58–2.19); however, when it comes to having a low or high dental caries score, having a normal BMI increases the chances of having a lower dental caries index by at least 10% (OR = 1.83, CI: 1.10–3.03) (Table 7).

## 4. Discussion

A comparison between the sexes revealed significantly higher levels of Vitamin D concentration and dental caries in boys, although age and BMI were not significantly different among the two groups. This might be due to sociocultural practices that allow boys to have more outdoor activities, consequently affecting serum Vitamin D levels. In addition, families may tolerate boys eating more sweets and enforce less oral hygiene on them.

When the three main variables were compared according to the season in which the data were collected, Vitamin D levels were significantly higher in summer in contrast to winter; a usual outcome that has been reported by many researchers [52,53]. On the other hand, BMI was higher in winter than in summer. This may reflect the cold weather of Sulaimani in winter, a season in which children tend to be relatively physically inactive and gain weight.

Interestingly enough, most of the children (70%) who participated in our research were vitamin D deficient. This result is especially dramatic since our study did not exclude individuals regularly taking Vitamin D supplements, i.e., the Vitamin D serum level is much lower in the majority of the population who do not receive supplements.

Despite Iraq’s favorable geographic position for the acquisition of sunlight throughout the year, the majority of the children in our survey were still Vitamin D deficient. These results agree with the findings of other Iraqi papers [54,55], as well as research from many different places worldwide [17,50].

Based on these high levels of deficiency among different populations globally, some authors argue that Vitamin D deficiency can be defined as an ‘epidemic’ [17]. In contrast, others find the normal range of ≥20 ng/mL to be non-realistic and call for the reduction of this value since most of the populations studied who are deficient are clinically healthy individuals [56]. The most prominent dispute in this field is the ongoing debate between the Institute of Medicine and the American Endocrine Society on the healthy normal range of Vitamin D serum levels [46].

Concerning the association between serum Vitamin D concentration and dental caries, the t-test value was insignificant (*p* = 0.22) (Table 4); however, surprisingly enough and contrary to other papers that reported a negative association [57,58], the association was positive. We believe this bias arises from the children on regular Vitamin D supplements. Hence, based on the recent developments in public health awareness about the essential role of Vitamin D in bone and dental health, we may hypothesize that children who have a higher caries index and suffer more from its consequences are given more supplements by their parents than children who suffer less from dental caries. This explanation is plausible, especially considering that the mean Vitamin D level was 15 ng/mL and 70% of the sample was deficient. Thus, we may argue that children with a Vitamin D level of ≥25 ng/mL were most probably on supplements, which falsely dragged the data toward a positive correlation.

When the data are treated as categorical, most of the CIs contained 0, which weakens the association, although the exact ORs of all our results were bigger than 1, except for the comparison between low- and high-caries groups with Vitamin D levels ≥15 ng/mL which revealed a significant OR of 1.88 (CI: 1.20–2.94), meaning that children with a sufficient level of Vitamin D have at least a 20% higher chance of having a lower caries index.

The majority of the participants had a normal BMI (70.4%), followed by 11.4% for underweight cases and 18.3% for overweight cases. This result is consistent with recent studies suggesting a decrease in malnutrition trends among Iraqi children [59,60]. As can be seen from our data, the prevalence of overweight children is higher than the underweight group. This is another interesting point that, in conjunction with other recent papers [61], shows that the classical distinction between developed and developing countries may no longer exist, as malnutrition appears in the form of undernutrition in developing countries while manifesting as obesity in developed ones. This may be due to a continuous increase in the modern industrialized lifestyle.

Moreover, in the linear regression, the correlation between BMI and DMFT was negative but insignificant (*p* = 0.55). However, in the categorized data, normal BMI was related to a lower caries index with an OR of 1.83 (CI: 1.10–3.03). This means that children with a normal BMI have 83% higher average chances of having lower dental caries. This particular result agrees with some papers [37] while contradicting others that suggest no relationship and/or are inconclusive [39,62].

Dental caries is a complex, multi-factorial, and chronic disease influenced mainly by oral hygiene, dietary habits, genetic predispositions, and regular check-ups. Unfortunately, due to the data collection process in a crowded hospital environment, this cross-sectional study could not measure the potential confounders. Further research is recommended to study this association, considering the potential covariates. Another limitation was the collection of underweight and overweight groups under the abnormal category since the focus of this study was on normal BMI, even though they may have different effects on dental caries.

## 5. Conclusions

Vitamin D deficiency is a serious issue, with the majority of our studied sample being at risk. Our results show that having ≥15 ng/mL of serum Vitamin D and having a normal BMI increase the chances of children having a lower caries index. Hence, practicing dentists should be able to identify the causes and risk factors of dental caries, among them modifying factors like Vitamin D deficiency and age-specific BMI. Public health policies targeting a healthy diet, sufficient outdoor activities, and oral hygiene are recommended.

## Figures and Tables

**Table 1:** Descriptive characteristics of the participants.

Sex (n/%)	Female	188	56.3
	Male	146	43.7
Age (year) (Median & IQR)		8	7–10
BMI (kg/m^2^) (Median & IQR)		15.7	14.5–17.8
BMI three categories (n/%)	Normal	235	70.4
	Underweight	38	11.4
	Overweight	61	18.3
BMI two categories (n/%)	Normal	234	70.3
	Abnormal	99	29.7
Vitamin D (ng/mL) (Median & IQR)		15.0	10.1–21.5
Vitamin D (ng/mL) (n/%) ≥ 20	Normal	100	30
	Deficient	233	70
Vitamin D (ng/mL) (n/%) ≥ 15	Normal	168	50.5
	Deficient	165	49.5
Season of the examinations (n/%)	Winter	128	38.3
	Summer	206	61.7
DMFT index (Median & IQR)		4	2–6
DMFT two categories (n/%)	Caries	286	85.9
	No Caries	47	14.1
DMFT two categories (n/%)	Low Caries (DMFT < 4)	203	61
	High caries (DMFT > 4)	130	39

BMI: Body Mass Index, DMFT: Decayed, Missed, and Filled Teeth, IQR: Inter-Quartile Range.

**Table 2:** Comparison of age, BMI, DMFT index, and serum Vitamin D levels between boys and girls.

	Female	Male		
	Median	Median	Z	*p*
Age (year)	8	8	0.142	0.887
BMI (kg/m^2^)	15.9	15.6	1.307	0.191
Vitamin D (ng/mL)	13.6	17.3	4.734	<0.001

BMI: Body mass index, DMFT: Decayed, Missing, and Filled Teeth, Z: Mann–Whitney U test.

**Table 3:** Comparison of BMI, DMFT index, and serum Vitamin D levels between the seasons.

	Winter	Summer		
	Median	Median	Z	*p*
BMI (kg/m^2^)	16.1	15.6	2.195	0.028
DMFT index	3	4	1.297	0.195
Vitamin D (ng/mL)	11.6	16.8	5.425	<0.001

BMI: Body mass index, DMFT: Decayed, Missing, and Filled Teeth, Z: Mann–Whitney U test.

**Table 4:** Regression analysis output showing the effects of age, sex, seasons, BMI, and vitamin D on DMFT.

	B	SE of B	t	*p*
Age	−0.37	0.094	−3.94	<0.001
Sex	0.58	0.34	1.69	0.09
Seasons	−0.005	0.35	−0.015	0.98
BMI	−0.03	0.05	−0.59	0.55
Vitamin D	0.025	0.021	1.21	0.22

BMI: Body mass index, DMFT: Decayed, Missing, and Filled Teeth, SE: Standard Error.

**Table 5:** Association between caries–no caries groups and serum vitamin D thresholds (≥20 ng/mL) (≥15 ng/mL).

	Serum Vitamin D (ng/mL)	Odds Ratio (95% of Confidence Intervals)
Caries Free (DMFT = 0)	≥20	1.97 (0.91–4.24)
Caries (DMFT > 0)	<20	
Caries Free (DMFT = 0)	≥15	1.60 (0.85–3)
Caries (DMFT > 0)	<15	

DMFT: Decayed, Missed and Filled Teeth.

**Table 6:** Association between low–high caries groups and serum vitamin D thresholds (≥20 ng/mL) (≥15 ng/mL).

	Serum Vitamin D (ng/mL)	Odds Ratio (95% of Confidence Intervals)
Low caries (DMFT < 4)	≥20	1.19 (0.74–1.92)
High caries (DMFT > 4)	<20	
Low caries (DMFT < 4)	≥15	1.88 (1.20–2.94)
High caries (DMFT > 4)	<15	

DMFT: Decayed, Missed and Filled Teeth.

**Table 7:** Association between dental caries and BMI.

	BMI	Odds Ratio (95% of Confidence Intervals)
Caries Free (DMFT = 0)	Normal	1.12 (0.58–2.19)
Caries (DMFT > 0)	Abnormal	
Low caries (DMFT < 4)	Normal	1.83 (1.10–3.03)
High caries (DMFT > 4	Abnormal	

BMI: Body Mass Index, DMFT: Decayed, Missed, and Filled Teeth.

## Data Availability

Not applicable.
